# 2-Bromo-1-(4-hydroxy­phen­yl)ethanone

**DOI:** 10.1107/S1600536809043013

**Published:** 2009-10-23

**Authors:** Wei-Xia Qing, Wei Zhang

**Affiliations:** a Medical College of Henan University, Henan University, Kaifeng 475004, People’s Republic of China; b Department of Pharmacy, Zhengzhou Railway Vocational and Technological College;, Zhengzhou 450052, People’s Republic of China

## Abstract

There are two mol­ecules in the asymmetric unit of the title compound, C_8_H_7_BrO_2_. In the crystal, they form independent chains propagating in [010] linked by O—H⋯O hydrogen bonds.

## Related literature

For medicinal background, see: Kumar *et al.* (1997 ▶).
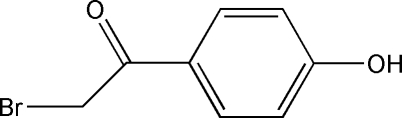


## Experimental

### 

#### Crystal data

C_8_H_7_BrO_2_
*M* *_r_* = 215.04
Monoclinic, 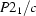

*a* = 8.6495 (15) Å
*b* = 15.052 (3) Å
*c* = 14.3562 (19) Å
β = 123.224 (7)°
*V* = 1563.5 (5) Å^3^
*Z* = 8
Mo *K*α radiation
μ = 5.20 mm^−1^
*T* = 293 K
0.38 × 0.34 × 0.29 mm

#### Data collection

Bruker SMART CCD diffractometer
Absorption correction: multi-scan (*SADABS*; Bruker, 2001 ▶) *T*_min_ = 0.243, *T*_max_ = 0.314
7916 measured reflections
3064 independent reflections
1752 reflections with *I* > 2σ(*I*)
*R*_int_ = 0.058

#### Refinement

*R*[*F* ^2^ > 2σ(*F* ^2^)] = 0.053
*wR*(*F* ^2^) = 0.142
*S* = 1.03
3064 reflections
199 parameters
H-atom parameters constrained
Δρ_max_ = 0.63 e Å^−3^
Δρ_min_ = −0.79 e Å^−3^

### 

Data collection: *SMART* (Bruker, 2001 ▶); cell refinement: *SAINT-Plus* (Bruker, 2001 ▶); data reduction: *SAINT-Plus*; program(s) used to solve structure: *SHELXS97* (Sheldrick, 2008 ▶); program(s) used to refine structure: *SHELXL97* (Sheldrick, 2008 ▶); molecular graphics: *PLATON* (Spek, 2009 ▶); software used to prepare material for publication: *PLATON*.

## Supplementary Material

Crystal structure: contains datablocks global, I. DOI: 10.1107/S1600536809043013/hb5143sup1.cif

Structure factors: contains datablocks I. DOI: 10.1107/S1600536809043013/hb5143Isup2.hkl

Additional supplementary materials:  crystallographic information; 3D view; checkCIF report

## Figures and Tables

**Table 1:** Hydrogen-bond geometry (Å, °)

*D*—H⋯*A*	*D*—H	H⋯*A*	*D*⋯*A*	*D*—H⋯*A*
O2—H2⋯O1^i^	0.82	2.02	2.811 (6)	162
O4—H4⋯O3^ii^	0.82	2.00	2.776 (5)	158

Symmetry codes: (i) 
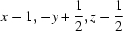
; (ii) 
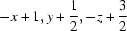
.

**Fig. 1.**
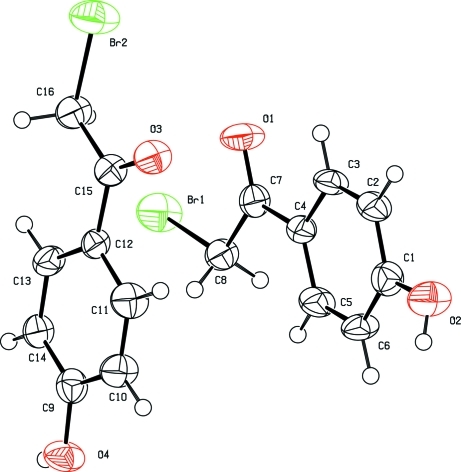
The molecular structure of (I), showing displacement ellipsoids drawn at the 50% probability level.

**Fig. 2.**
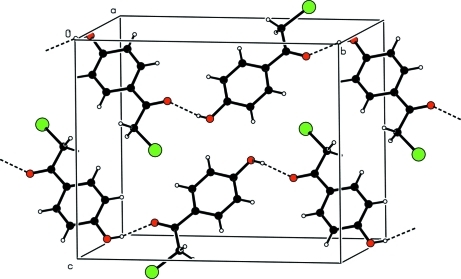
One-dimensional structure of (I) along [010] direction, Hydrogen bonds are shown as dashed lines.

## supplementary crystallographic information

### Comment

The title compound, (I), 2-bromo-1-(4-hydroxyphenyl)ethanone is widely used in the synthesis of adrenaline-type drugs (e.g. Kumar *et al.*,  1997). Herein, we report the crystal structure of the title compound (I).

As shown in Fig.1, the title compound (I) consists of an asymmetric organic molecule. The S(6) ring of C(1)/C(2)/C(3)/C(4)/C(5)/C(6) in (I) is an aromatic ring. In the structure, C(7)–O(1) [1.212 (7) Å] and C(15)–O(3) [1.203 (6) Å] is typical for a C==O double bond, whereas, the C(1)–O(2), and C(9)–O(4) bond distances are of 1.349 (7) and 1.355 (7) Å, respectively (Table 1), indicating two obviously C–O single bonds.

In the crystal structure, these molecules are linked into infinite one-dimensional chains by intermolecular O—H···O hydrogen bonds running along [010] direction (Fig. 2, Table 1).

### Experimental

4-Hydroxyacetophenone (10 g, 73.4 mmol) was dissolved in chloroform (50 ml) at 338 K. With stirring, concentrated sulfuric acid (3.80 ml, 1.84 g/ml) was added in the solution. After stirring for 10 min, bromine (3.9 ml, 76.1 mmol) was added in reaction solution. After 5 h, the solution was quenched with water (60 ml), the layers were separated and the aqueous layer was extracted with chloroform, the combined organic extracts were washed with saturated aqueous sodium bicarbonate solution (30 ml), dried (MgSO_4_) and evaporated under reduced pressure to give the crude product. Then purification by short column chromatography (chloroform) and recrystallization from chloroform gave the compound (I) as orange blocks (12.79 g, 81%).

### Refinement

H atoms were geometrically placed (C—H = 0.93–0.97Å, O—H = 0.82 Å) and refined as riding with *U*_iso_(*H*)=1.2*U*_eq_(C) or *U*_iso_(H)=1.5*U*_eq_(O).

### Crystal data

**Table:**

C_8_H_7_BrO_2_	*F*(000) = 848
*M**_r_* = 215.04	*D*_x_ = 1.827 Mg m^−^^3^
Monoclinic, *P*2_1_/*c*	Mo *K*α radiation, λ = 0.71073 Å
Hall symbol: -P 2ybc	Cell parameters from 1628 reflections
*a* = 8.6495 (15) Å	θ = 2.7–23.1°
*b* = 15.052 (3) Å	µ = 5.20 mm^−^^1^
*c* = 14.3562 (19) Å	*T* = 293 K
β = 123.224 (7)°	Block, orange
*V* = 1563.5 (5) Å^3^	0.38 × 0.34 × 0.29 mm
*Z* = 8	

### Data collection

**Table:**

Bruker SMART CCD diffractometer	3064 independent reflections
Radiation source: fine-focus sealed tube	1752 reflections with *I* > 2σ(*I*)
graphite	*R*_int_ = 0.058
ω scans	θ_max_ = 26.0°, θ_min_ = 2.7°
Absorption correction: multi-scan (*SADABS*; Bruker, 2001)	*h* = −5→10
*T*_min_ = 0.243, *T*_max_ = 0.314	*k* = −18→18
7916 measured reflections	*l* = −17→17

### Refinement

**Table:**

Refinement on *F*^2^	Primary atom site location: structure-invariant direct methods
Least-squares matrix: full	Secondary atom site location: difference Fourier map
*R*[*F*^2^ > 2σ(*F*^2^)] = 0.053	Hydrogen site location: inferred from neighbouring sites
*wR*(*F*^2^) = 0.142	H-atom parameters constrained
*S* = 1.03	*w* = 1/[σ^2^(*F*_o_^2^) + (0.0679*P*)^2^ + 0.3873*P*] where *P* = (*F*_o_^2^ + 2*F*_c_^2^)/3
3064 reflections	(Δ/σ)_max_ = 0.001
199 parameters	Δρ_max_ = 0.63 e Å^−^^3^
0 restraints	Δρ_min_ = −0.79 e Å^−^^3^

### Special details

**Table:**

Geometry. All e.s.d.'s (except the e.s.d. in the dihedral angle between two l.s. planes) are estimated using the full covariance matrix. The cell e.s.d.'s are taken into account individually in the estimation of e.s.d.'s in distances, angles and torsion angles; correlations between e.s.d.'s in cell parameters are only used when they are defined by crystal symmetry. An approximate (isotropic) treatment of cell e.s.d.'s is used for estimating e.s.d.'s involving l.s. planes.
Refinement. Refinement of *F*^2^ against ALL reflections. The weighted *R*-factor *wR* and goodness of fit *S* are based on *F*^2^, conventional *R*-factors *R* are based on *F*, with *F* set to zero for negative *F*^2^. The threshold expression of *F*^2^ > σ(*F*^2^) is used only for calculating *R*-factors(gt) *etc*. and is not relevant to the choice of reflections for refinement. *R*-factors based on *F*^2^ are statistically about twice as large as those based on *F*, and *R*- factors based on ALL data will be even larger.

### Fractional atomic coordinates and isotropic or equivalent isotropic displacement parameters (Å^2^)

**Table:**

	*x*	*y*	*z*	*U*_iso_*/*U*_eq_	
Br1	0.93107 (10)	0.53760 (4)	0.71841 (6)	0.0609 (3)	
O1	0.9475 (6)	0.3392 (2)	0.7394 (4)	0.0546 (12)	
O2	0.2872 (6)	0.0898 (2)	0.4104 (3)	0.0546 (12)	
H2	0.1818	0.1092	0.3705	0.082*	
C1	0.4050 (9)	0.1575 (4)	0.4654 (5)	0.0412 (15)	
C2	0.5763 (8)	0.1372 (4)	0.5584 (5)	0.0401 (15)	
H2A	0.6070	0.0781	0.5797	0.048*	
C3	0.7014 (8)	0.2024 (3)	0.6197 (5)	0.0371 (14)	
H3A	0.8141	0.1872	0.6837	0.044*	
C4	0.6631 (8)	0.2913 (3)	0.5878 (4)	0.0326 (13)	
C5	0.4910 (9)	0.3105 (4)	0.4922 (5)	0.0426 (15)	
H5A	0.4612	0.3693	0.4695	0.051*	
C6	0.3633 (9)	0.2450 (4)	0.4300 (5)	0.0409 (15)	
H6A	0.2509	0.2594	0.3653	0.049*	
C7	0.7990 (9)	0.3582 (3)	0.6569 (5)	0.0375 (14)	
C8	0.7466 (9)	0.4533 (3)	0.6211 (5)	0.0398 (15)	
H8A	0.6330	0.4665	0.6167	0.048*	
H8B	0.7223	0.4603	0.5470	0.048*	
Br2	1.06321 (10)	0.28455 (5)	1.05320 (6)	0.0680 (3)	
O3	0.6801 (6)	0.2668 (2)	0.8479 (3)	0.0446 (11)	
O4	0.1884 (6)	0.5955 (2)	0.5816 (3)	0.0489 (11)	
H4	0.2337	0.6450	0.5896	0.073*	
C9	0.3201 (9)	0.5383 (4)	0.6552 (5)	0.0393 (15)	
C10	0.2821 (9)	0.4486 (4)	0.6349 (5)	0.0456 (16)	
H10A	0.1697	0.4297	0.5734	0.055*	
C11	0.4103 (8)	0.3883 (4)	0.7055 (5)	0.0445 (16)	
H11A	0.3822	0.3281	0.6922	0.053*	
C12	0.5815 (8)	0.4134 (3)	0.7966 (4)	0.0310 (13)	
C13	0.6156 (9)	0.5033 (4)	0.8149 (5)	0.0391 (14)	
H13A	0.7271	0.5224	0.8770	0.047*	
C14	0.4887 (9)	0.5657 (4)	0.7435 (5)	0.0388 (15)	
H14A	0.5174	0.6259	0.7552	0.047*	
C15	0.7123 (8)	0.3449 (4)	0.8668 (5)	0.0357 (14)	
C16	0.8958 (8)	0.3773 (4)	0.9621 (5)	0.0430 (15)	
H16A	0.8753	0.4158	1.0085	0.052*	
H16B	0.9534	0.4126	0.9326	0.052*	

### Atomic displacement parameters (Å^2^)

**Table:**

	*U*^11^	*U*^22^	*U*^33^	*U*^12^	*U*^13^	*U*^23^
Br1	0.0530 (5)	0.0346 (4)	0.0735 (5)	−0.0082 (3)	0.0209 (4)	−0.0036 (3)
O1	0.035 (3)	0.035 (2)	0.050 (3)	0.003 (2)	−0.005 (3)	0.000 (2)
O2	0.042 (3)	0.033 (2)	0.060 (3)	−0.009 (2)	0.010 (2)	−0.004 (2)
C1	0.044 (4)	0.038 (4)	0.043 (4)	−0.001 (3)	0.024 (4)	−0.003 (3)
C2	0.044 (4)	0.026 (3)	0.042 (4)	0.008 (3)	0.018 (4)	0.006 (3)
C3	0.032 (3)	0.027 (3)	0.040 (3)	0.007 (3)	0.012 (3)	0.005 (3)
C4	0.033 (3)	0.029 (3)	0.030 (3)	0.005 (3)	0.014 (3)	0.002 (2)
C5	0.045 (4)	0.026 (3)	0.047 (4)	0.007 (3)	0.019 (4)	0.005 (3)
C6	0.038 (4)	0.027 (3)	0.037 (3)	0.004 (3)	0.007 (3)	0.006 (3)
C7	0.043 (4)	0.028 (3)	0.039 (4)	−0.004 (3)	0.021 (4)	−0.005 (3)
C8	0.044 (4)	0.027 (3)	0.038 (3)	0.000 (3)	0.015 (3)	0.000 (2)
Br2	0.0486 (5)	0.0459 (4)	0.0771 (6)	0.0048 (3)	0.0138 (4)	0.0072 (3)
O3	0.052 (3)	0.018 (2)	0.047 (2)	−0.0043 (19)	0.017 (2)	0.0005 (17)
O4	0.043 (3)	0.027 (2)	0.057 (3)	0.005 (2)	0.015 (2)	0.0044 (19)
C9	0.045 (4)	0.035 (3)	0.040 (4)	0.000 (3)	0.025 (4)	−0.001 (3)
C10	0.039 (4)	0.032 (3)	0.050 (4)	−0.008 (3)	0.014 (3)	−0.005 (3)
C11	0.045 (4)	0.024 (3)	0.054 (4)	−0.005 (3)	0.021 (4)	−0.001 (3)
C12	0.036 (4)	0.023 (3)	0.029 (3)	−0.002 (3)	0.015 (3)	−0.002 (2)
C13	0.037 (4)	0.034 (3)	0.033 (3)	−0.001 (3)	0.010 (3)	−0.004 (3)
C14	0.043 (4)	0.022 (3)	0.041 (4)	−0.004 (3)	0.016 (4)	−0.009 (3)
C15	0.038 (4)	0.033 (3)	0.036 (3)	−0.002 (3)	0.020 (3)	0.001 (3)
C16	0.046 (4)	0.027 (3)	0.042 (4)	−0.003 (3)	0.016 (4)	0.001 (3)

### Geometric parameters (Å, °)

**Table:**

Br1—C8	1.915 (6)	Br2—C16	1.918 (6)
O1—C7	1.212 (7)	O3—C15	1.203 (6)
O2—C1	1.349 (7)	O4—C9	1.355 (7)
O2—H2	0.8200	O4—H4	0.8199
C1—C2	1.380 (8)	C9—C14	1.370 (9)
C1—C6	1.387 (8)	C9—C10	1.382 (8)
C2—C3	1.366 (8)	C10—C11	1.360 (8)
C2—H2A	0.9300	C10—H10A	0.9300
C3—C4	1.395 (7)	C11—C12	1.387 (7)
C3—H3A	0.9300	C11—H11A	0.9300
C4—C5	1.395 (8)	C12—C13	1.379 (8)
C4—C7	1.449 (8)	C12—C15	1.453 (7)
C5—C6	1.381 (8)	C13—C14	1.380 (8)
C5—H5A	0.9300	C13—H13A	0.9300
C6—H6A	0.9300	C14—H14A	0.9300
C7—C8	1.504 (7)	C15—C16	1.500 (8)
C8—H8A	0.9700	C16—H16A	0.9700
C8—H8B	0.9700	C16—H16B	0.9700
			
C1—O2—H2	109.5	C9—O4—H4	109.6
O2—C1—C2	117.7 (5)	O4—C9—C14	122.9 (5)
O2—C1—C6	122.8 (6)	O4—C9—C10	117.1 (6)
C2—C1—C6	119.4 (6)	C14—C9—C10	119.9 (6)
C3—C2—C1	121.0 (5)	C11—C10—C9	119.5 (6)
C3—C2—H2A	119.5	C11—C10—H10A	120.3
C1—C2—H2A	119.5	C9—C10—H10A	120.3
C2—C3—C4	121.1 (5)	C10—C11—C12	122.2 (5)
C2—C3—H3A	119.4	C10—C11—H11A	118.9
C4—C3—H3A	119.4	C12—C11—H11A	118.9
C5—C4—C3	117.1 (5)	C13—C12—C11	117.0 (5)
C5—C4—C7	123.9 (5)	C13—C12—C15	124.1 (5)
C3—C4—C7	119.0 (5)	C11—C12—C15	118.9 (5)
C6—C5—C4	122.2 (5)	C12—C13—C14	121.7 (6)
C6—C5—H5A	118.9	C12—C13—H13A	119.1
C4—C5—H5A	118.9	C14—C13—H13A	119.1
C5—C6—C1	119.0 (6)	C9—C14—C13	119.5 (5)
C5—C6—H6A	120.5	C9—C14—H14A	120.2
C1—C6—H6A	120.5	C13—C14—H14A	120.2
O1—C7—C4	122.2 (5)	O3—C15—C12	122.7 (5)
O1—C7—C8	121.2 (5)	O3—C15—C16	121.4 (5)
C4—C7—C8	116.6 (5)	C12—C15—C16	115.7 (5)
C7—C8—Br1	114.0 (4)	C15—C16—Br2	114.3 (4)
C7—C8—H8A	108.8	C15—C16—H16A	108.7
Br1—C8—H8A	108.8	Br2—C16—H16A	108.7
C7—C8—H8B	108.8	C15—C16—H16B	108.7
Br1—C8—H8B	108.8	Br2—C16—H16B	108.7
H8A—C8—H8B	107.6	H16A—C16—H16B	107.6

### Hydrogen-bond geometry (Å, °)

**Table:**

*D*—H···*A*	*D*—H	H···*A*	*D*···*A*	*D*—H···*A*
O2—H2···O1^i^	0.82	2.02	2.811 (6)	162
O4—H4···O3^ii^	0.82	2.00	2.776 (5)	158

Symmetry codes: (i) *x*−1, −*y*+1/2, *z*−1/2; (ii) −*x*+1, *y*+1/2, −*z*+3/2.
